# 1-(4-Meth­oxy­phen­yl)-2-(1*H*-1,2,4-triazol-1-yl)ethanone

**DOI:** 10.1107/S160053681002653X

**Published:** 2010-07-10

**Authors:** Victor Kesternich, Iván Brito, Michael Bolte, Marcia Pérez-Fermann, Ronald Nelson

**Affiliations:** a Departamento de Química, Universidad Católica del Norte, Casilla 1280, Antofagasta, Chile; b Departamento de Química, Facultad de Ciencias Básicas, Universidad de Antofagasta, Casilla 170, Antofagasta, Chile; c Institut für Anorganische Chemie der Goethe-Universität Frankfurt, Max-von-Laue-Strasse 7, D-60438 Frankfurt am Main, Germany

## Abstract

In the title compound, C_11_H_11_N_3_O_2_, the dihedral angle between the central ethanone fragment and the 4-meth­oxy­phenyl group is 2.9 (2)°, while that between the ethanone fragment and the triazole ring is 83.4 (2)°. The dihedral angle between the planes of the triazole and benzene rings is 81.7 (1)°. The 4-meth­oxy­phenyl group is *cis* with respect to the ethanone fragment O atom across the exocyclic C—C bond. In the crystal, mol­ecules are linked by C—H⋯N inter­actions into *C*(9) chains along [001].

## Related literature

For the biological activity of fungal infections, see: Wingard & Leather (2004 ▶); Lamb *et al.* (1999 ▶). For the synthesis, see: Emami *et al.* (2008 ▶); Upadhayaya *et al.* (2009 ▶); Schiaffella *et al.* (2005 ▶); Dawood *et al.* (2006 ▶). For hydrogen-bond motifs, see: Bernstein *et al.* (1995 ▶).
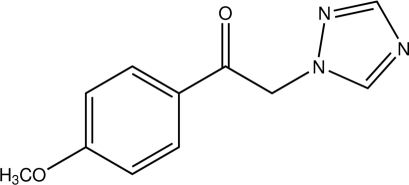


## Experimental

### 

#### Crystal data

C_11_H_11_N_3_O_2_
*M* *_r_* = 217.23
Monoclinic, 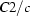

*a* = 23.409 (3) Å
*b* = 4.8347 (7) Å
*c* = 20.607 (2) Å
β = 116.275 (8)°
*V* = 2091.2 (5) Å^3^
*Z* = 8
Mo *K*α radiation
μ = 0.10 mm^−1^
*T* = 173 K
0.29 × 0.25 × 0.21 mm

#### Data collection

Stoe IPDS II two-circle diffractometer
4675 measured reflections
1944 independent reflections
1260 reflections with *I* > 2σ(*I*)
*R*_int_ = 0.053

#### Refinement

*R*[*F* ^2^ > 2σ(*F* ^2^)] = 0.041
*wR*(*F* ^2^) = 0.101
*S* = 0.89
1944 reflections
147 parameters
H-atom parameters constrained
Δρ_max_ = 0.16 e Å^−3^
Δρ_min_ = −0.16 e Å^−3^

### 

Data collection: *X-AREA* (Stoe & Cie, 2001 ▶); cell refinement: *X-AREA*; data reduction: *X-AREA*; program(s) used to solve structure: *SHELXS97* (Sheldrick, 2008 ▶); program(s) used to refine structure: *SHELXL97* (Sheldrick, 2008 ▶); molecular graphics: *XP* (Sheldrick, 2008 ▶); software used to prepare material for publication: *SHELXL97*.

## Supplementary Material

Crystal structure: contains datablocks I, global. DOI: 10.1107/S160053681002653X/fl2306sup1.cif

Structure factors: contains datablocks I. DOI: 10.1107/S160053681002653X/fl2306Isup2.hkl

Additional supplementary materials:  crystallographic information; 3D view; checkCIF report

## Figures and Tables

**Table 1:** Hydrogen-bond geometry (Å, °)

*D*—H⋯*A*	*D*—H	H⋯*A*	*D*⋯*A*	*D*—H⋯*A*
C15—H15⋯N4^i^	0.95	2.42	3.336 (3)	162

Symmetry code: (i) 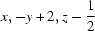.

**Fig. 1.**
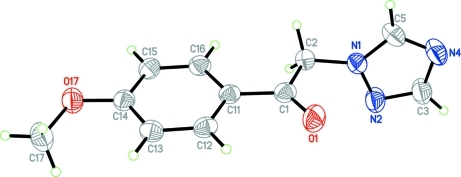
Perspective view of (I) with the atom numbering; displacement ellipsoids are at the 50% probability level (arbitrary spheres for the H atoms).

**Fig. 2.**
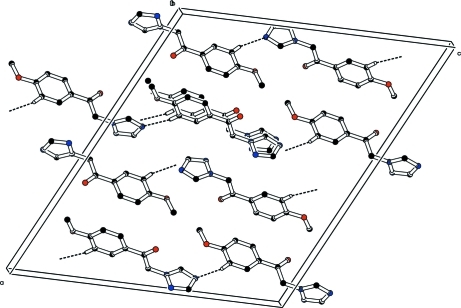
Packing diagram for (I) showing the formation of a C(9) chain along [001]. Hydrogen bond shown as dashed lines.

**Fig. 3.**
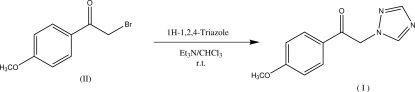
The formation of the title compound.

## supplementary crystallographic information

### Comment

Fungal infections caused by pathogenic species, often characterized by high mortality rates, has been increasing over the past two decades. In the treatment of fungal infections the number of efficacious antifungal drugs is limited (Wingard & Leather, 2004). Many of the currently available drugs are toxic, produce recurrence because they are fungistatic and not fungicides or lead to the development of resistance due in part to the prolonged periods of administration of the available antifungal drugs (Lamb *et al.*, 1999). In order to seek new antifungal agents we are preparing a series of substituted triazoles, fluconazole analogues (Emami *et al.*, 2008).

In this article we report the synthesis and crystal structure of the titl compound, (I). In (I), Fig. 1, the dihedral angle between the central OCC ethanone fragment and the *o*-methoxyphenyl group is 2.9 (2)°, while that with group triazole is 83.4 (2)°. The dihedral angle between the plane of triazole and benzene ring is 81.7 (1)°. The *o*-methoxyphenyl group is *cis* with respect to the ethanone fragment O atom across the C11—C1 bond. In the crystal molecules are linked by C—H···N interactions into chains with graph-set notation C(9) along [001] (Bernstein *et al.*, 1995), Table 1, Fig. 2.

### Experimental

Compound (II), was synthesized as described by Upadhayaya, *et al.*, (2009). Compound (I) was synthesized from (II) as described by Schiaffella *et al.*, (2005) and Dawood *et al.*, (2006) as shown in scheme 1. Recrystallization of (I) from methanol/chloroform (9/1) at room temperature afforded colourless crystals suitable for X-ray diffraction analysis.

### Refinement

All H atoms could be located by difference Fourier synthesis but were ultimately placed in calculated positions using a riding model with C—H(aromatic) = 0.95 Å, *C*—*H*(methylene) = 0.99 Å and *C*—*H*(methyl) = 0.98 Å with fixed individual displacement parameters [*U*_iso_(H) = 1.2 *U*_eq_(C) or 1.5 *U*_eq_(C_methyl_)].

### Crystal data

**Table:**

C_11_H_11_N_3_O_2_	*F*(000) = 912
*M**_r_* = 217.23	*D*_x_ = 1.380 Mg m^−^^3^
Monoclinic, *C*2/*c*	Mo *K*α radiation, λ = 0.71073 Å
Hall symbol: -C 2yc	Cell parameters from 3087 reflections
*a* = 23.409 (3) Å	θ = 3.6–25.9°
*b* = 4.8347 (7) Å	µ = 0.10 mm^−^^1^
*c* = 20.607 (2) Å	*T* = 173 K
β = 116.275 (8)°	Block, colourless
*V* = 2091.2 (5) Å^3^	0.29 × 0.25 × 0.21 mm
*Z* = 8	

### Data collection

**Table:**

Stoe IPDS II two-circle diffractometer	1260 reflections with *I* > 2σ(*I*)
Radiation source: fine-focus sealed tube	*R*_int_ = 0.053
graphite	θ_max_ = 25.6°, θ_min_ = 3.5°
ω scans	*h* = −27→28
4675 measured reflections	*k* = −5→5
1944 independent reflections	*l* = −24→24

### Refinement

**Table:**

Refinement on *F*^2^	Secondary atom site location: difference Fourier map
Least-squares matrix: full	Hydrogen site location: inferred from neighbouring sites
*R*[*F*^2^ > 2σ(*F*^2^)] = 0.041	H-atom parameters constrained
*wR*(*F*^2^) = 0.101	*w* = 1/[σ^2^(*F*_o_^2^) + (0.0547*P*)^2^] where *P* = (*F*_o_^2^ + 2*F*_c_^2^)/3
*S* = 0.89	(Δ/σ)_max_ < 0.001
1944 reflections	Δρ_max_ = 0.16 e Å^−^^3^
147 parameters	Δρ_min_ = −0.16 e Å^−^^3^
0 restraints	Extinction correction: *SHELXL97* (Sheldrick, 2008), Fc^*^=kFc[1+0.001xFc^2^λ^3^/sin(2θ)]^-1/4^
Primary atom site location: structure-invariant direct methods	Extinction coefficient: 0.0060 (8)

### Special details

**Table:**

Geometry. All e.s.d.'s (except the e.s.d. in the dihedral angle between two l.s. planes) are estimated using the full covariance matrix. The cell e.s.d.'s are taken into account individually in the estimation of e.s.d.'s in distances, angles and torsion angles; correlations between e.s.d.'s in cell parameters are only used when they are defined by crystal symmetry. An approximate (isotropic) treatment of cell e.s.d.'s is used for estimating e.s.d.'s involving l.s. planes.
Refinement. Refinement of *F*^2^ against ALL reflections. The weighted *R*-factor *wR* and goodness of fit *S* are based on *F*^2^, conventional *R*-factors *R* are based on *F*, with *F* set to zero for negative *F*^2^. The threshold expression of *F*^2^ > σ(*F*^2^) is used only for calculating *R*-factors(gt) *etc*. and is not relevant to the choice of reflections for refinement. *R*-factors based on *F*^2^ are statistically about twice as large as those based on *F*, and *R*- factors based on ALL data will be even larger.

### Fractional atomic coordinates and isotropic or equivalent isotropic displacement parameters (Å^2^)

**Table:**

	*x*	*y*	*z*	*U*_iso_*/*U*_eq_	
O1	0.34820 (7)	0.4726 (3)	0.43819 (7)	0.0493 (4)	
C1	0.37507 (9)	0.6336 (4)	0.41489 (9)	0.0347 (4)	
C2	0.42565 (9)	0.8304 (4)	0.46522 (9)	0.0358 (4)	
H2A	0.4651	0.8043	0.4595	0.043*	
H2B	0.4110	1.0230	0.4512	0.043*	
N1	0.43985 (8)	0.7900 (3)	0.54036 (8)	0.0355 (4)	
N2	0.47973 (9)	0.5846 (4)	0.57991 (8)	0.0521 (5)	
C3	0.47828 (12)	0.6132 (5)	0.64287 (10)	0.0512 (6)	
H3	0.5022	0.4971	0.6830	0.061*	
N4	0.44080 (9)	0.8176 (4)	0.64643 (8)	0.0486 (5)	
C5	0.41747 (10)	0.9240 (4)	0.58069 (10)	0.0428 (5)	
H5	0.3887	1.0753	0.5644	0.051*	
C11	0.36082 (8)	0.6444 (4)	0.33756 (9)	0.0307 (4)	
C12	0.31663 (9)	0.4624 (4)	0.28896 (9)	0.0344 (4)	
H12	0.2954	0.3354	0.3060	0.041*	
C13	0.30274 (9)	0.4612 (4)	0.21646 (9)	0.0353 (4)	
H13	0.2724	0.3351	0.1841	0.042*	
C14	0.33387 (9)	0.6477 (4)	0.19143 (8)	0.0335 (4)	
C15	0.37810 (9)	0.8315 (4)	0.23879 (9)	0.0366 (4)	
H15	0.3993	0.9581	0.2215	0.044*	
C16	0.39130 (9)	0.8305 (4)	0.31086 (9)	0.0354 (4)	
H16	0.4215	0.9577	0.3430	0.042*	
O17	0.32364 (7)	0.6657 (3)	0.12113 (6)	0.0433 (4)	
C17	0.28122 (11)	0.4693 (5)	0.07117 (10)	0.0513 (6)	
H17A	0.2951	0.2817	0.0893	0.077*	
H17B	0.2815	0.4918	0.0240	0.077*	
H17C	0.2380	0.4998	0.0659	0.077*	

### Atomic displacement parameters (Å^2^)

**Table:**

	*U*^11^	*U*^22^	*U*^33^	*U*^12^	*U*^13^	*U*^23^
O1	0.0624 (10)	0.0507 (9)	0.0465 (8)	−0.0140 (8)	0.0348 (7)	−0.0002 (6)
C1	0.0380 (10)	0.0301 (9)	0.0422 (9)	0.0052 (9)	0.0235 (8)	0.0035 (8)
C2	0.0422 (11)	0.0346 (10)	0.0345 (9)	0.0008 (9)	0.0205 (8)	0.0003 (7)
N1	0.0396 (9)	0.0362 (9)	0.0349 (7)	0.0056 (7)	0.0202 (7)	0.0004 (6)
N2	0.0676 (13)	0.0570 (11)	0.0409 (8)	0.0258 (10)	0.0325 (9)	0.0113 (8)
C3	0.0627 (14)	0.0583 (13)	0.0397 (10)	0.0182 (12)	0.0290 (10)	0.0059 (10)
N4	0.0523 (11)	0.0584 (11)	0.0425 (9)	0.0069 (9)	0.0278 (8)	−0.0072 (8)
C5	0.0439 (12)	0.0443 (12)	0.0424 (10)	0.0044 (10)	0.0210 (9)	−0.0097 (9)
C11	0.0313 (10)	0.0272 (9)	0.0374 (8)	0.0038 (8)	0.0187 (8)	0.0020 (7)
C12	0.0342 (10)	0.0297 (9)	0.0434 (9)	−0.0003 (8)	0.0209 (8)	0.0033 (8)
C13	0.0334 (10)	0.0331 (10)	0.0385 (9)	−0.0021 (8)	0.0151 (8)	−0.0028 (7)
C14	0.0352 (10)	0.0330 (10)	0.0343 (9)	0.0061 (8)	0.0172 (8)	0.0030 (7)
C15	0.0419 (11)	0.0330 (10)	0.0390 (9)	−0.0042 (9)	0.0214 (8)	0.0035 (7)
C16	0.0380 (11)	0.0308 (10)	0.0391 (9)	−0.0040 (8)	0.0187 (8)	−0.0010 (7)
O17	0.0515 (9)	0.0453 (8)	0.0334 (6)	−0.0071 (7)	0.0192 (6)	−0.0014 (6)
C17	0.0568 (14)	0.0580 (13)	0.0361 (9)	−0.0122 (11)	0.0178 (10)	−0.0087 (9)

### Geometric parameters (Å, °)

**Table:**

O1—C1	1.225 (2)	C11—C16	1.403 (2)
C1—C11	1.477 (2)	C12—C13	1.381 (2)
C1—C2	1.515 (3)	C12—H12	0.9500
C2—N1	1.446 (2)	C13—C14	1.394 (2)
C2—H2A	0.9900	C13—H13	0.9500
C2—H2B	0.9900	C14—O17	1.3624 (19)
N1—C5	1.330 (2)	C14—C15	1.386 (3)
N1—N2	1.360 (2)	C15—C16	1.377 (2)
N2—C3	1.320 (2)	C15—H15	0.9500
C3—N4	1.345 (3)	C16—H16	0.9500
C3—H3	0.9500	O17—C17	1.429 (2)
N4—C5	1.320 (2)	C17—H17A	0.9800
C5—H5	0.9500	C17—H17B	0.9800
C11—C12	1.389 (3)	C17—H17C	0.9800
			
O1—C1—C11	122.36 (18)	C13—C12—C11	121.74 (16)
O1—C1—C2	120.80 (15)	C13—C12—H12	119.1
C11—C1—C2	116.83 (14)	C11—C12—H12	119.1
N1—C2—C1	112.82 (14)	C12—C13—C14	119.11 (17)
N1—C2—H2A	109.0	C12—C13—H13	120.4
C1—C2—H2A	109.0	C14—C13—H13	120.4
N1—C2—H2B	109.0	O17—C14—C15	115.64 (15)
C1—C2—H2B	109.0	O17—C14—C13	124.08 (17)
H2A—C2—H2B	107.8	C15—C14—C13	120.28 (15)
C5—N1—N2	109.70 (14)	C16—C15—C14	119.90 (16)
C5—N1—C2	129.47 (17)	C16—C15—H15	120.0
N2—N1—C2	120.79 (14)	C14—C15—H15	120.0
C3—N2—N1	101.70 (15)	C15—C16—C11	121.00 (18)
N2—C3—N4	115.60 (18)	C15—C16—H16	119.5
N2—C3—H3	122.2	C11—C16—H16	119.5
N4—C3—H3	122.2	C14—O17—C17	117.55 (14)
C5—N4—C3	102.33 (15)	O17—C17—H17A	109.5
N4—C5—N1	110.67 (18)	O17—C17—H17B	109.5
N4—C5—H5	124.7	H17A—C17—H17B	109.5
N1—C5—H5	124.7	O17—C17—H17C	109.5
C12—C11—C16	117.97 (15)	H17A—C17—H17C	109.5
C12—C11—C1	119.73 (15)	H17B—C17—H17C	109.5
C16—C11—C1	122.29 (17)		
			
O1—C1—C2—N1	3.8 (2)	C2—C1—C11—C16	−1.2 (3)
C11—C1—C2—N1	−175.44 (15)	C16—C11—C12—C13	0.2 (3)
C1—C2—N1—C5	−96.6 (2)	C1—C11—C12—C13	−178.68 (17)
C1—C2—N1—N2	80.8 (2)	C11—C12—C13—C14	0.0 (3)
C5—N1—N2—C3	−0.3 (2)	C12—C13—C14—O17	−179.61 (17)
C2—N1—N2—C3	−178.21 (18)	C12—C13—C14—C15	0.0 (3)
N1—N2—C3—N4	0.3 (3)	O17—C14—C15—C16	179.50 (17)
N2—C3—N4—C5	−0.2 (3)	C13—C14—C15—C16	−0.1 (3)
C3—N4—C5—N1	0.0 (2)	C14—C15—C16—C11	0.3 (3)
N2—N1—C5—N4	0.1 (2)	C12—C11—C16—C15	−0.4 (3)
C2—N1—C5—N4	177.86 (19)	C1—C11—C16—C15	178.51 (17)
O1—C1—C11—C12	−1.6 (3)	C15—C14—O17—C17	176.40 (18)
C2—C1—C11—C12	177.64 (16)	C13—C14—O17—C17	−4.0 (3)
O1—C1—C11—C16	179.50 (18)		

### Hydrogen-bond geometry (Å, °)

**Table:**

*D*—H···*A*	*D*—H	H···*A*	*D*···*A*	*D*—H···*A*
C15—H15···N4^i^	0.95	2.42	3.336 (3)	162

Symmetry codes: (i) *x*, −*y*+2, *z*−1/2.
